# Inversion of the uterus with placenta adherens and successful reposition

**DOI:** 10.1007/s00404-021-06175-8

**Published:** 2021-08-10

**Authors:** Anne Dathan-Stumpf, Bahriye Aktas, Laura Weydandt, Holger Stepan

**Affiliations:** 1 Department of Obstetrics, University Hospital Leipzig, Liebigstrasse 20a, 04103 Leipzig, Germany; 2 Department of Gynecology, University Hospital Leipzig, Liebigstrasse 20a, 04103 Leipzig, Germany

**Keywords:** Inversio uteri, Placenta adherens, Treatment, Reposition, Complication in childbirth

## Description

**Fig. 1:**
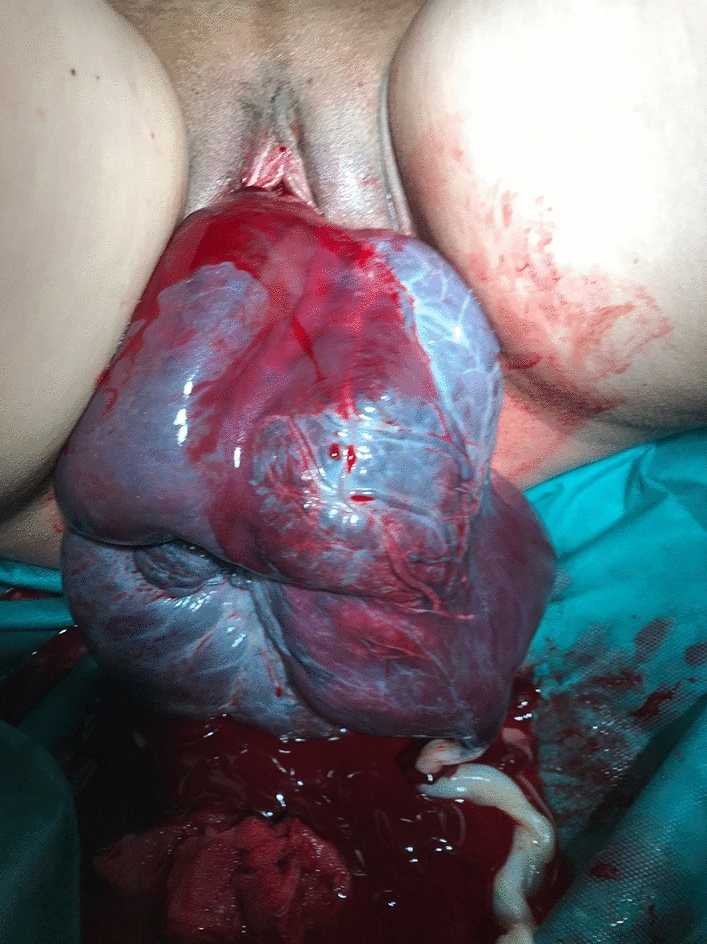
Inverted uterus with adherent placenta

**Fig. 2:**
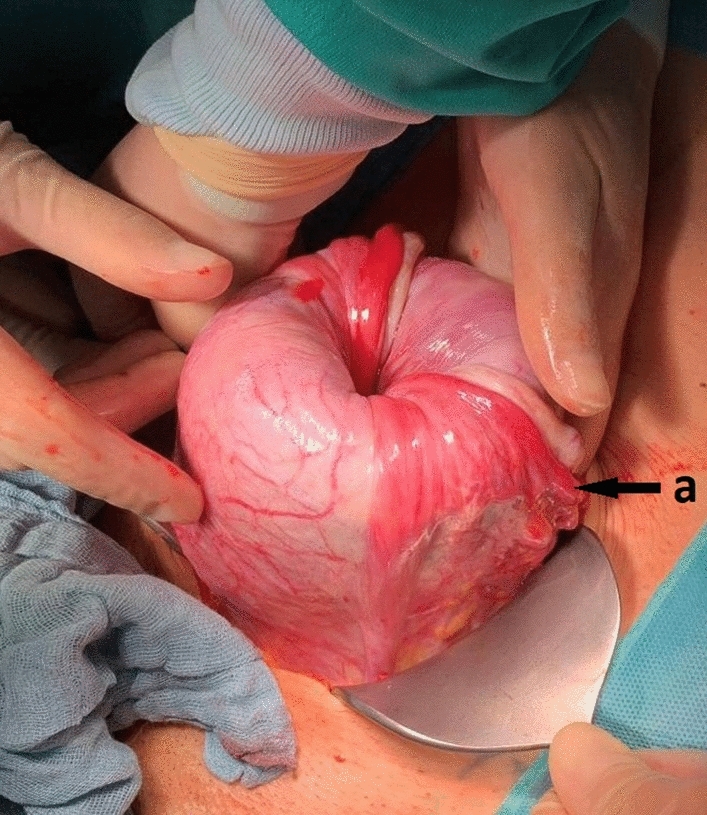
Repositioned uterus with still inverted fundus and visible fimbriae of the tube (**a**)

**Fig. 3:**
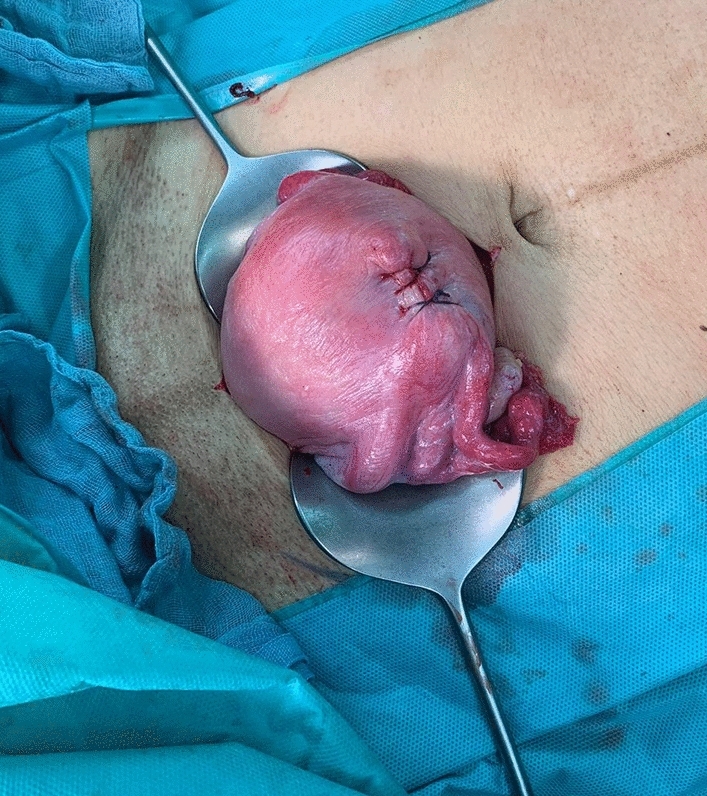
Normal configured uterus after complete repositioning and suturing

A 33-year-old second para had an uneventful vaginal delivery of a female newborn (3240 g) in the 39th week of gestation. Oxytocin was given and 30 min after delivery, cord traction was performed to remove the placenta. The inverted uterus with the adherent placenta prolapsed (Fig. 1). The woman was immediately transferred into the operation room and manual replacement was attempted in general anaesthesia. Placenta could be separated, but laparotomy was necessary to correct the uterine fundus that was still inverted (Fig. 2). Only the tubal fimbriae were visible (Fig. 2a). The complete repositioning of the fundus was succeeded by manual pressure with the hand from vaginally. An iatrogenic lesion was repaired with two sutures (Fig. 3).

Postpartum haemorrhage with a total blood loss of 3000 millilitres was treated with oxytocin, prostaglandins, fluid and blood transfusion. One day after delivery, the uterus showed a normal configuration and was contracted well.

## Data Availability

Not applicable.

